# Lupus Nephritis and Hydroxychloroquine-Associated Zebra Bodies: Not Just in Fabry Disease

**DOI:** 10.1016/j.xkme.2021.01.006

**Published:** 2021-03-18

**Authors:** Shun Manabe, Toshio Mochizuki, Masayo Sato, Hiroshi Kataoka, Sekiko Taneda, Kazuho Honda, Keiko Uchida, Kosaku Nitta

**Affiliations:** 1 Department of Nephrology, Tokyo Women’s Medical University, Tokyo, Japan; 2 Clinical Research Division for Polycystic Kidney Disease, Department of Medicine, Kidney Center, Tokyo Women’s Medical University, Tokyo, Japan; 3 Department of Pathology, Tokyo Women’s Medical University, Tokyo, Japan; 4 Department of Anatomy, Showa University School of Medicine, Tokyo, Japan

**Keywords:** Chloroquine, Fabry disease, hydroxychloroquine, lupus nephritis, phospholipidosis, zebra body

## Abstract

Zebra bodies in kidney biopsy specimens are widely accepted as a specific feature of Fabry disease but they can also be present in a drug-induced mimic of Fabry disease, phospholipidosis. Chloroquine and hydroxychloroquine may both induce zebra body formation and kidney phospholipidosis. However, the frequency and clinical significance of such changes remain unknown. We report 5 serial kidney biopsy cases diagnosed as lupus nephritis during hydroxychloroquine administration. All 5 patients exhibited a few, but varying amounts, of zebra bodies in glomerular intrinsic cells, that is, podocytes, parietal epithelial cells, mesangial cells, and endothelial cells. Most of the zebra bodies detected were subtle, though certainly recognizable; these zebra bodies were much smaller than those observed in Fabry disease. Zebra bodies were not observed in patients with lupus nephritis in the absence of chloroquine or hydroxychloroquine administration. All patients with lupus nephritis who received hydroxychloroquine achieved complete remission during continuous use of hydroxychloroquine, though kidney toxicity of drug-induced phospholipidosis might be masked by immunosuppression. Based on this small series of cases, we speculate that the hydroxychloroquine-associated manifestation of zebra bodies and phospholipidosis in the kidney may be frequent phenomena and may have only a subclinical influence on kidney function, at least in the short term.

## Introduction

Chloroquine and hydroxychloroquine, which were first developed as antimalarial agents, are now used widely for treating systemic lupus erythematosus because of their numerous immunomodulatory functions.^1^^,^^2^ Chloroquine and hydroxychloroquine ameliorate systemic lupus erythematosus by suppressing the activation of Toll-like receptors that are expressed on the surface of endosomes.^1^^,^^2^ At the same time, chloroquine and hydroxychloroquine accumulate in lysosomes, resulting in inhibition of the endosome-lysosome pathway and autophagic flux.^2^^,^^3^ Chloroquine and hydroxychloroquine are effective for systemic lupus erythematosus treatment; however, some serious adverse effects, such as retinopathy, myopathy, and cardiomyopathy, have been reported.^1^^,^^2^^,^^4^^,^^5^ The mechanism underlying the adverse effects of chloroquine and hydroxychloroquine is associated with their accumulation in lysosomes, which increases their intravesicular pH, resulting in lysosomal enzyme dysfunction and metabolite accumulation, namely phospholipids.^1^^,^^2^^,^^3^^,^^6^ Experiments investigating chloroquine-associated phospholipidosis have demonstrated the accumulation of zebra bodies and phospholipids in systemic organs.^4^ Biopsy tissues of patients exhibiting myopathy and cardiomyopathy contain zebra bodies.^5^ Therefore, the emergence of zebra bodies is assumed to be a sign of chloroquine- and hydroxychloroquine-induced lysosomal dysfunction, which may result in metabolite accumulation and organ damage.

Kidneys are one of the main target organs of systemic lupus erythematosus. Chloroquine and hydroxychloroquine protect against kidney damage in lupus nephritis.^1^^,^^2^^,^^7^ Although rare, zebra body accumulation in the kidney has been reported in patients treated with chloroquine and hydroxychloroquine^6^ (Table S1^8^, ^9^, ^10^, ^11^, ^12^, ^13^, ^14^, ^15^). Detection of such unusual intracellular structures requires differential diagnosis from Fabry disease, a condition that is typically characterized by zebra bodies. After the exclusion of Fabry disease, potentially useful chloroquine and hydroxychloroquine treatment should be discontinued to avoid organ damage caused by phospholipidosis. However, the prevalence and clinical significance of chloroquine- and hydroxychloroquine-associated zebra bodies in the kidney remain unknown.

We report 5 serial kidney biopsy cases diagnosed as lupus nephritis during hydroxychloroquine treatment. All biopsies contained varying amounts of zebra bodies. These results suggested that zebra body formation and kidney phospholipidosis may be frequently associated with hydroxychloroquine administration.

## Case report

Clinical characteristics of the patients at the time of the kidney biopsies and their clinical courses are summarized in Tables 1 and S2. None of the patients had a family history or manifested symptoms associated with Fabry disease (Table S2). In detail, none of the patients had a history of transient ischemic attack or stroke or were aware of neuropathic pain or hearing loss. Electrocardiograms were normal in all patients, and echocardiogram testing in 3 patients did not show apparent thickening of the intraventricular septum and posterior left ventricular wall. Dermatologic assessment indicated no angiokeratoma, and ophthalmic assessment indicated no cornea verticillata. The indications of the biopsies were nephritis (3 patients) and nephrotic syndrome (2 patients). Durations of hydroxychloroquine treatment were 10 days to 4 years. Daily and cumulative dosages of hydroxychloroquine were 4.3 to 7.2 mg/kg of body weight and 3 to 576 g, respectively. Immunosuppressants selected for induction therapy were prednisolone and mycophenolate mofetil in 4 patients and prednisolone, mycophenolate mofetil, and tacrolimus in 1 patient. All patients continued hydroxychloroquine treatment after the kidney biopsy. Urinary protein levels improved in all 5 patients, reaching <0.3 g/g creatinine in 4 patients and <0.5 g/g creatinine in 1 patient.

**Figure 1:**
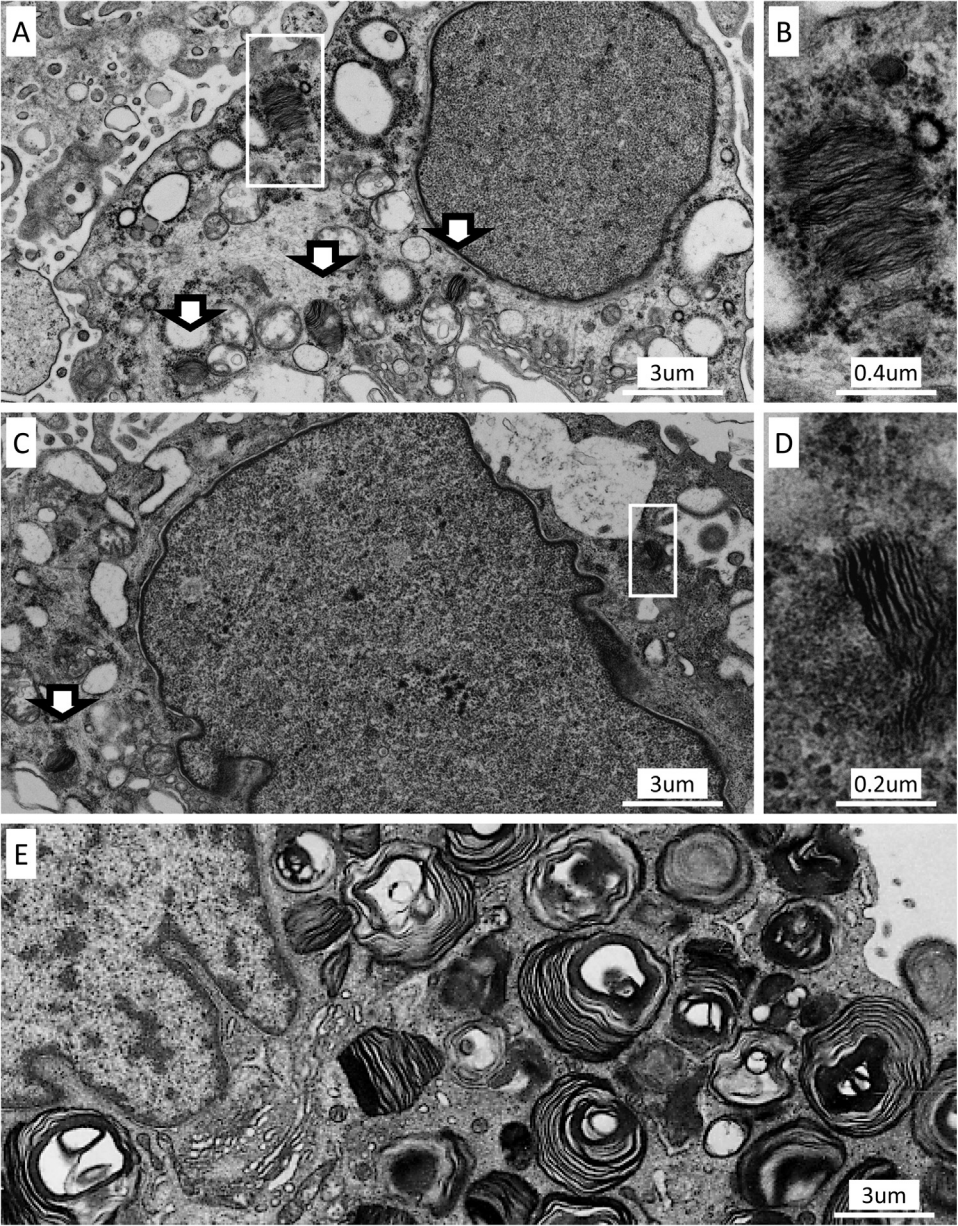
Ultrastructural findings of the kidney biopsies. (A, B) Patient 3. (A) Podocyte with several zebra bodies (arrowed and framed box around). The maximum number of zebra bodies in a single podocyte was 6 in this patient. (B) High-magnification image of the boxed zebra body. (C, D) Patient 2. (C) Podocyte with subtle zebra bodies (arrowed and framed box around). Most zebra bodies in hydroxychloroquine-treated patients were like these and difficult to detect, though certainly recognizable in a high-magnification image. (D) High-magnification image of the boxed zebra body. (E) Podocyte from a male patient with Fabry disease shows numerous zebra bodies. (A, C, E) Presented in the same magnification, though the size and number of zebra bodies differ between (A, C) hydroxychloroquine-treated patients and (E) patient with Fabry disease.

The following diagnoses were made based on biopsies: lupus nephritis class III (2two patients), class IV-S (2 patients), and class IV-S + V (1 patient; Table 1). Zebra bodies were apparent in all 5 patients; 4 in podocytes, 1 in parietal epithelial cells, 3 in mesangial cells, and 1 in endothelial cells. Zebra bodies were not detected in 14 patients with lupus nephritis who were not treated with chloroquine and hydroxychloroquine diagnosed in the same period. Patient 3 exhibited the most prominent zebra bodies (Fig 1A and B). Up to 6 zebra bodies per single cell were observed in multiple podocytes. However, most of the zebra bodies detected were subtle, although certainly recognizable (patient 2, Fig 1C and D). The number of zebra bodies was much lower in hydroxychloroquine-associated patients than in 4 male patients with Fabry disease diagnosed at our institute (Fig 1E). The maximum number of zebra bodies per single cell was 2 to 6 (average, 3.6) in hydroxychloroquine-associated cases, but 59 to 141 (average, 89.5) in Fabry disease cases. In addition, the “size” of zebra bodies was small in hydroxychloroquine-associated cases (Fig 1A and C) compared with Fabry disease cases (Fig 1E).

**Table 1:** Clinical Characteristics and Kidney Biopsy Findings of Present Cases

Case	Age, y/Sex	Diagnosis	HCQ Doses per BW, mg/kg	Duration of HCQ Treatment at Biopsy, mo	Cumulative HCQ Dose at Biopsy, g	Urinary Protein at Biopsy, g/g Cr	Scr at Biopsy, mg/dL	Diagnosis of Kidney Biopsy	Treatment	Observation Period After Biopsy, mo	Urinary Protein After Biopsy, g/g Cr	Scr After Biopsy, mg/dL	Maximum No. of Zebra Bodies per Cell; Cell Type; No.
1	34/F	SLE	5.9	8	63	0.35	0.54	III(A/C)	PSL, MMF, HCQ	8	0.17	0.78	Mes; 2
2	51/F	SLE	5.5	28	243	2.9	0.77	IV-S(A/C)	PSL, MMF, HCQ	8	0.25	0.74	Pod; 3, Endo; 1
3	20/F	SLE, SjS	4.3	4	27	1.2	0.49	IV-S(A/C)	PSL, MMF, HCQ	5	0	0.41	Pod; 6, Mes; 1
4	34/F	SLE	7.2	48	576	9.1	0.52	IV-G(A/C)+V	PSL, MMF, Tac, HCQ	13	0.49	0.69	Pod; 5, PEC; 1, Endo 1
5	42/F	SLE, SjS, APS	5.0	0.3	3	0.45	0.71	III(A/C)	PSL, MMF, HCQ	11	0	0.73	Mes; 2, Pod; 1

Abbreviations: APS, antiphospholipid syndrome; BW, body weight; Endo, endothelial cell; F, female; HCQ, hydroxychloroquine; Mes, mesangial cell; MMF, mycophenolate mofetil’ PEC, parietal epithelial cell; Pod, podocyte; PSL, prednisolone; SjS, Sjögren syndrome; SLE, systemic lupus erythematosus; Tac, tacrolimus.

Vesicles with osmiophilic granules were observed in the fragmented podocytes in 2 cases (Fig S1A) and in proximal tubular epithelial cells in all cases (Fig S1B). These osmiophilic granules were surrounded by a single-unit membrane, which indicated their presence in lysosome-associated pathways. Curvilinear body, another unusual intracellular structure that emerges in phospholipidosis,^8^^,^^9^^,^^12^ was not observed.

## Discussion

We report 5 serial kidney biopsies of patients with lupus nephritis during hydroxychloroquine treatment. All patients exhibited zebra bodies, and to our knowledge, this is the first report of their high prevalence. However, the number and size of zebra bodies formed during hydroxychloroquine treatment were far smaller than those in patients with Fabry disease. These findings might be consistent with a previous review,^6^^, p. 242^ which specified that “podocyte inclusions are more numerous in Fabry disease.” Clinically, all patients continued hydroxychloroquine treatment and achieved complete remission. This makes it difficult to determine the clinical significance of hydroxychloroquine-associated zebra bodies in lupus nephritis.

Chloroquine and hydroxychloroquine are cationic amphiphilic drugs that have an affinity for components of the endosome-lysosome pathway and autophagic flux because of their relatively low electron potential.^1^, ^2^, ^3^ They accumulate in lysosomes, thereby increasing their intravesicular pH, and inhibit various lysosomal enzymes, including α-galactosidase and phospholipases A and C.^1^, ^2^, ^3^ Alpha-galactosidase is the causative enzyme for Fabry disease. Absence or reduced α-galactosidase activity results in the accumulation of glycosphingolipids, a subtype of glycolipids, which ultrastructurally form zebra bodies.^6^^,^^8^, ^9^, ^10^, ^11^, ^12^, ^13^, ^14^, ^15^ Phospholipases A and C metabolize phospholipids, and their reduced activity results in an accumulation of osmiophilic phospholipids.^8^^,^^10^^,^^13^^,^^16^ Therefore, inhibition of these enzymes by chloroquine and hydroxychloroquine is presumed to result in the formation of zebra bodies and osmiophilic granules.

In previous studies investigating zebra bodies during chloroquine or hydroxychloroquine treatment by kidney biopsy, enzyme activity or genetic analyses were performed in 7 of the 8 patients 8, 9, 10, 11, 12, 13, 14, 15 (Table S1). All tested patients did not have Fabry disease.^8^, ^9^, ^10^, ^11^, ^12^^,^^14^^,^^15^ In 1 patient who did not undergo enzyme activity or genetic analyses, a repeat kidney biopsy was performed after the cession of hydroxychloroquine treatment, which indicated the disappearance of zebra bodies.^13^ In our cases, the patients lacked family histories and symptoms suggestive of Fabry disease, including cornea verticillata, which is reported to be observed in most women with Fabry disease.^17^ Further, based on the small number and size of the zebra bodies, a likely diagnosis of hydroxychloroquine-associated phospholipidosis was made.^6^ However, because genetic analysis is the only method to confirm the diagnosis of female Fabry disease and Fabry disease shows various phenotypic variants, genetic analysis should be considered if possible.

Histologic differences between hydroxychloroquine-associated kidney phospholipidosis and Fabry disease remain to be clarified. The existence of curvilinear bodies has been reported to be a distinguishing factor.^5^^,^^8^^,^^9^^,^^12^ However, not all previously reported cases^10^^,^^11^^,^^13^, ^14^, ^15^ and none of the present cases exhibited curvilinear bodies. Another histologic finding was the presence of intracellular vesicles with osmiophilic granules in the cytoplasm of some podocytes (Fig S1A) and all proximal tubular epithelial cells (Fig S1B). In our cases, these vesicles containing osmiophilic granules were detected in only hydroxychloroquine-associated cases, but not in lupus nephritis without chloroquine and hydroxychloroquine use or Fabry disease. We speculate that these osmiophilic granules represent phospholipids accumulated due to drug-induced lysosomal enzyme disfunction.^8^^,^^9^^,^^16^ Moreover, another cationic amphiphilic drug, amiodarone, is reported to cause zebra bodies and vesicles containing osmiophilic granules.^18^^,^^19^ Because tubular secretion eliminates 40% to 70% of orally administered chloroquine and hydroxychloroquine,^1^^,^^20^ it is likely that these drugs accumulate in proximal tubular epithelial cells, resulting in further lysosomal dysfunction. In contrast to rare and small zebra bodies in glomeruli, vesicles containing osmiophilic granules in proximal tubular epithelial cells might easily distinguish hydroxychloroquine-associated phospholipidosis from Fabry disease.

The clinical significance of hydroxychloroquine-associated zebra bodies and kidney phospholipidosis is another unclarified issue. Of the 8 patients—in previous reports—who exhibited zebra bodies during chloroquine and hydroxychloroquine treatment^8^, ^9^, ^10^, ^11^, ^12^, ^13^, ^14^, ^15^ (Table S1), only 1 showed apparent improvement in kidney function on suspension of chloroquine treatment alone.^8^ Three patients with suspended chloroquine or hydroxychloroquine therapy did not show apparent improvement.^10^, ^11^, ^12^ In 1 patient, in whom improvement of proteinuria might have been associated with angiotensin-converting enzyme inhibitor administration, the zebra bodies disappeared after ceasing hydroxychloroquine treatment.^13^

These previous reports suggest that the zebra bodies—a sign of phospholipid accumulation—do not necessarily decrease kidney function. In our cases, the formation of a small number of tiny zebra bodies might be a subclinical phenomenon, at least in the short term, though its kidney toxicity might be masked by immunosuppression. Because chloroquine and hydroxychloroquine can effectively prevent systemic lupus erythematosus flare-ups and prevent kidney damage in lupus nephritis,^1^^,^^2^^,^^7^ continuous administration of these drugs might benefit patients even if the kidney biopsy reveals zebra body formation and phospholipidosis.

In summary, we report 5 patients with lupus nephritis treated with hydroxychloroquine who exhibited a small number of tiny zebra bodies and kidney phospholipidosis. Manifestation of hydroxychloroquine-associated zebra bodies might be a frequent and subclinical phenomenon. This suggests that it is not necessary to cease potentially beneficial hydroxychloroquine therapy. Further studies are required to determine the true prevalence and clinical significance of hydroxychloroquine-associated zebra bodies and kidney phospholipidosis.
