# Supplementary material for: Lupus Nephritis and Hydroxychloroquine-Associated Zebra Bodies: Not Just in Fabry Disease
Source: Kidney Med. 2021 Mar 18;3(3):442–6. doi: 10.1016/j.xkme.2021.01.006 (PMC8178476; doi:10.1016/j.xkme.2021.01.006)
Supplement: Supplementary File (PDF) — Figure S1; Tables S1-S2. [file mmc1.pdf]

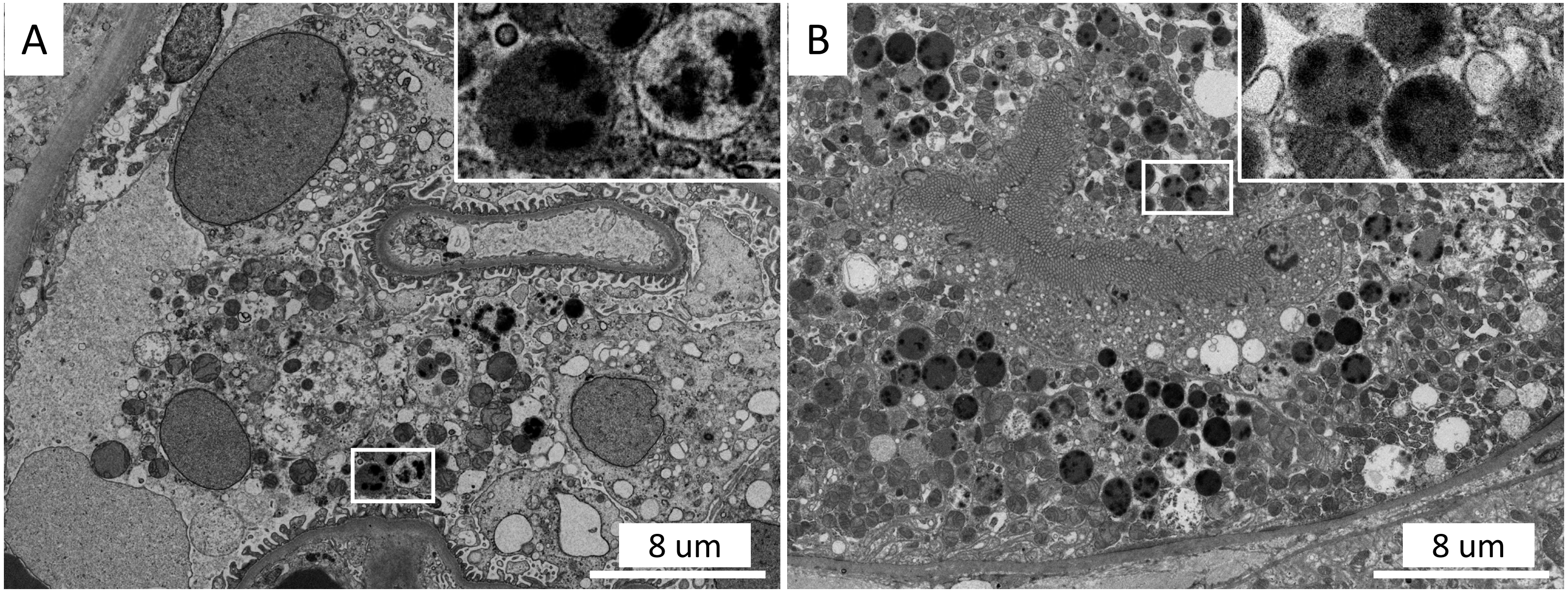

Supplementary Figure 1. Ultrastructural findings of the kidney biopsy. Osmiophilic granules of patient III. (A) Fragmented podocyte with intracellular vesicles including osmiophilic granules (x1,500, inset x6,000). (B) Swollen proximal tubular epithelial cells with intracellular vesicles including osmiophilic granules (x1,500, inset x6,000).

Supplementary table 1. Clinical characteristics and kidney biopsy findings of previously reported cases.

Case	Age Sex	Diagnosis	CQ or HCQ	CQ/HCQ dose (mg/day)	Duration of CQ/HCQ (year)	Indication for KBx	Urine protein at KBx (g/day)	Serum creatinine at KBx (mg/dL)	GLA activity	GLA activity (normal range)	*GLA* Mutation	Zebra-like body	Curvilinear body	Treatment	Clinical outcome	Year [Ref]
1	46 F	SiS	CQ	155	0.9	kidney dysfunction	1.0	5.0	56	33.2-109	ND	+	+	CQ suspension	improved	2003 [8]
2	56 F	RA	CQ	500	1.5	kidney dysfunction	trace	2.2	33.2	8.8-42.6	none	+	+	CQ and NSAIDs suspension	unchanged	2005 [9]
3	56 F	arteritis	HCQ	ND	10	proteinuria	3.3	ND	normal		none	+	-	HCQ suspension ACEI	unchanged	2006 [10]
4	70 F	SLE	HCQ	ND	8	proteinuria	2	ND	normal		none	+	-	HCQ suspension	unchanged	2007 [11]
5	31 F	SLE	HCQ	150	3	initiation for belimumab	0.6	ND	8.18	2.1-13.6	none	+	+	HCQ suspension	unchanged	2013 [12]
6	28 F	SLE	HCQ	400	1.2	proteinuria	0.6	ND	ND		ND	+	-	HCQ suspension Thalidomide ACEI	improved	2017 [13]
7	26 F	SLE	HCQ	ND	9	nephrotic syndrome	6.9	ND	normal		none	+	-	HCQ suspension IVCY	Improved	2017 [14]
8	27 F	SLE	HCQ	ND	15	proteinuria	0.4-3.8	ND	ND		none	+	-	ND	ND	2018 [15]

CQ; chloroquine, HCQ; hydroxychloroquine, KBx; kidney biopsy, GLA; alpha-galactosidase, Ref; Number of reference, F; female, SjS; Sjogren's syndrome, RA; rheumatoid arthritis, SLE; systemic lupus erythematosus, ND; not documented, NSAIDs; nonsteroidal anti-inflammatory drugs, ACEI; angiotensin converting enzyme inhibitor, IVCY; intravenous cyclophosphamide,

Supplementary table 2. Clinical characteristics, kidney biopsy findings and assessment for Fabry disease of the present cases.

Case	Age Sex	Diagnosis	CQ or HCQ	CQ/HCQ dose (mg/day)	Duration of CQ/HCQ (year)	Indication for KBx	Urine protein at KBx (g/day)	Serum creatinine at KBx (mg/dL)	GLA activity	*GLA* Mutation	Zebra -like body	Curvilinear body	Treatment	Clinical outcome	Electro-cardiogram	Echo-cardiogram	Neuro-pathic pain	Cornea verticillata
I	34 F	SLE	HCQ	300	0.7	recurrence of nephritis	0.35	0.54	NA	NA	+	-	PSL, MMF, HCQ	improved	normal	NA	-	-
II	51 F	SLE	HCQ	300	2.3	nephrotic syndrome	2.9	0.77	NA	NA	+	-	PSL, MMF, HCQ	improved	normal	IVST 10mm PWT 9mm	-	-
III	20 F	SLE, SjS	HCQ	200	0.3	nephritis	1.2	0.49	NA	NA	+	-	PSL, MMF, HCQ	improved	normal	IVST 6mm PWT 6mm	-	-
IV	34 F	SLE	HCQ	400	4	recurrence of nephrotic syndrome	9.1	0.52	NA	NA	+	-	PSL, MMF, Tac, HCQ	improved	normal	NA	-	-
V	42 F	SLE, SiS, APS	HCQ	300	0.03	nephritis	0.45	0.71	NA	NA	+	-	PSL, MMF, HCQ	improved	normal	IVST 9mm PWT 9mm	-	-

CQ; chloroquine, HCQ; hydroxychloroquine, KBx; kidney biopsy, GLA; alpha-galactosidase, F; female, SLE; systemic lupus erythematosus, , NA; not analyzed, ISVT; intraventricular septum thickness, PWT; posterior left ventricular wall thickness, PSL; prednisolone, MMF; mycophenolate mofetil, SjS; Sjogren’s syndrome, Tac; tacrolimus, APS; anti-phospholipid syndrome
